# The impact of circadian rhythms on the immune response to influenza vaccination in middle-aged and older adults (IMPROVE): a randomised controlled trial

**DOI:** 10.1186/s12979-022-00304-w

**Published:** 2022-10-17

**Authors:** Yihao Liu, Hui Zhang, Gang Yuan, Mi Yao, Bin Li, Jianying Chen, Yuling Fan, Ruohui Mo, Fenghua Lai, Xinwen Chen, Mengyuan Li, Binfeng Chen, Janet M. Lord, Sui Peng, KarKeung Cheng, Haipeng Xiao

**Affiliations:** 1 Clinical Trials Unit, The First Affiliated Hospital of Sun Yat-Sen University, No. 58, ZhongShan Road 2, Guangzhou, Guangdong 510080 People’s Republic of China; 2 Department of Endocrinology, The First Affiliated Hospital of Sun Yat-Sen University, No. 58, ZhongShan Road 2, Guangzhou, Guangdong 510080 People’s Republic of China; 3 Department of Rheumatology and Clinical Immunology, The First Affiliated Hospital of Sun Yat-Sen University, Guangzhou, People’s Republic of China; 4 Institute of Precision Medicine, The First Affiliated Hospital of Sun Yat-Sen University, Guangzhou, People’s Republic of China; 5 Phase I Clinical Trial Center, The First Affiliated Hospital of Sun Yat-Sen University, Guangzhou, People’s Republic of China; 6 Department of Geriatrics, The First Affiliated Hospital of Sun Yat-Sen University, Guangzhou, People’s Republic of China; 7 Institute of Applied Health Research, University of Birmingham, Public Health Building, Edgbaston, Birmingham, B15 2TT UK; 8 Baiyun Street Community Health Service Center, Guangzhou, 510080 People’s Republic of China; 9 Shipai Street Community Health Service Center, Guangzhou, 510080 People’s Republic of China; 10 Institute of Inflammation and Ageing, University of Birmingham, Birmingham, UK; 11 NIHR Birmingham Biomedical Research Centre, University Hospital Birmingham and University of Birmingham, Birmingham, UK

**Keywords:** Circadian, Immunity, Influenza vaccination, Older adults, Ageing

## Abstract

**Background:**

Vaccination is important in influenza prevention but the immune response wanes with age. The circadian nature of the immune system suggests that adjusting the time of vaccination may provide an opportunity to improve immunogenicity. Our previous cluster trial in Birmingham suggested differences between morning and afternoon vaccination for some strains in the influenza vaccine in older adults. Whether this effect is also seen in a younger age group with less likelihood of compromised immunity is unknown. We therefore conducted an individual-based randomized controlled trial in Guangzhou to test the hypothesis that influenza vaccination in the morning induces a stronger immune response in older adults than afternoon vaccination. We included adults in middle age to determine if the effect was also seen in younger age groups.

**Results:**

Of the 418 participants randomised, 389 (93.1%, 191 middle-aged adults aged 50–60 years and 198 older adults aged 65–75 years) were followed up. Overall, there was no significant difference between the antibody titers (geometric mean /95% CI) after morning vs afternoon vaccination (A/H1N1: 39.9 (32.4, 49.1) vs. 33.0 (26.7, 40.7), *p* = 0.178; A/H3N2: 92.2 (82.8, 102.7) vs. 82.0 (73.8, 91.2), *p* = 0.091; B: 15.8 (13.9, 17.9) vs. 14.4 (12.8, 16.3), *p* = 0.092), respectively. However, in pre-specified subgroup analyses, post-vaccination titers for morning versus afternoon vaccination in the 65–75 years subgroup were (A/H1N1): 49.5 (36.7, 66.6) vs. 32.9 (24.7, 43.9), *p* = 0.050; (A/H3N2): 93.5 (80.6, 108.5) vs. 73.1 (62.9, 84.9), *p *= 0.021; (B): 16.6 (13.8, 20.1) vs. 14.4 (12.3, 17.0), *p *= 0.095, respectively. Among females, antibody titers for morning versus afternoon vaccination were (A/H1N1): 46.9 (35.6, 61.8) vs. 31.1 (23.8, 40.7), *p* = 0.030; (A/H3N2): 96.0 (83.5, 110.3) vs. 84.7 (74.4, 96.5), *p* = 0.176; (B): 14.8 (12.7, 17.3) vs. 13.0 (11.3, 14.9), *p* = 0.061, respectively. In the 50–60 years old subgroup and males, there were no significant differences between morning and afternoon vaccination.

**Conclusions:**

Morning vaccination may enhance the immunogenicity to influenza vaccine in adults aged over 65 and women. An intervention to modify vaccination programs to vaccinate older individuals in the morning is simple, cost free and feasible in most health systems.

**Supplementary Information:**

The online version contains supplementary material available at 10.1186/s12979-022-00304-w.

## Introduction

Seasonal influenza virus infections cause substantial morbidity and mortality globally [1] and vaccination is one of the most effective preventive measures against influenza [2, 3]. As older adults account for the highest proportion of influenza-related hospitalizations and mortality [3, 4], they are a priority group for influenza vaccination. However, age-related decline in immunity impairs antibody responses to vaccines in older adults [5]. Despite the use of adjuvants, varied delivery methods and modified dosages to improve immunogenicity and clinical effectiveness, the effect of influenza vaccination remains suboptimal among older adults [6–8]. Effective interventions to enhance immune responses to vaccines among older adults would therefore have clinical and public health significance. This is especially relevant in the future influenza seasons when countries also face the prospect of endemic corona virus disease 2019 (COVID-19) and repeat waves of infection.

The immune system is significantly influenced by circadian rhythms, for example immune cells vary in cell number and function in a circadian fashion [9]. A small observational study [10] reported differences between immune response after morning vs afternoon influenza vaccination. Our previous trial in Birmingham [11] also suggested differences between morning and afternoon vaccination for some strains in the influenza vaccine. However the trial had several important limitations: first, it was a cluster trial with some differences in key baseline characteristics; second, as the study was conducted over three influenza seasons because of difficulties in recruitment, six different types of vaccine were used that included different strains each year; third, baseline blood samples were taken immediately before vaccination, meaning that there were systematic differences in baseline levels between morning and afternoon vaccination groups.

If there are meaningful differences in immune response to vaccination at different times of the day, this may represent a simple and low-cost intervention that can be accommodated in most settings. We therefore conducted an individual-based randomized controlled trial to test the hypothesis that influenza vaccination in the morning induces stronger immune response in older adults than afternoon vaccination. We also included adults in middle age to determine if any effect of time of day was also seen in a younger group with less likelihood of compromised immunity. In addition, as a previous study had also reported sex differences in the circadian responses to both hepatitis A and influenza vaccination [10], sex was also considered in our sub-group analysis.

## Results

### Participant characteristics

**Fig. 1:**
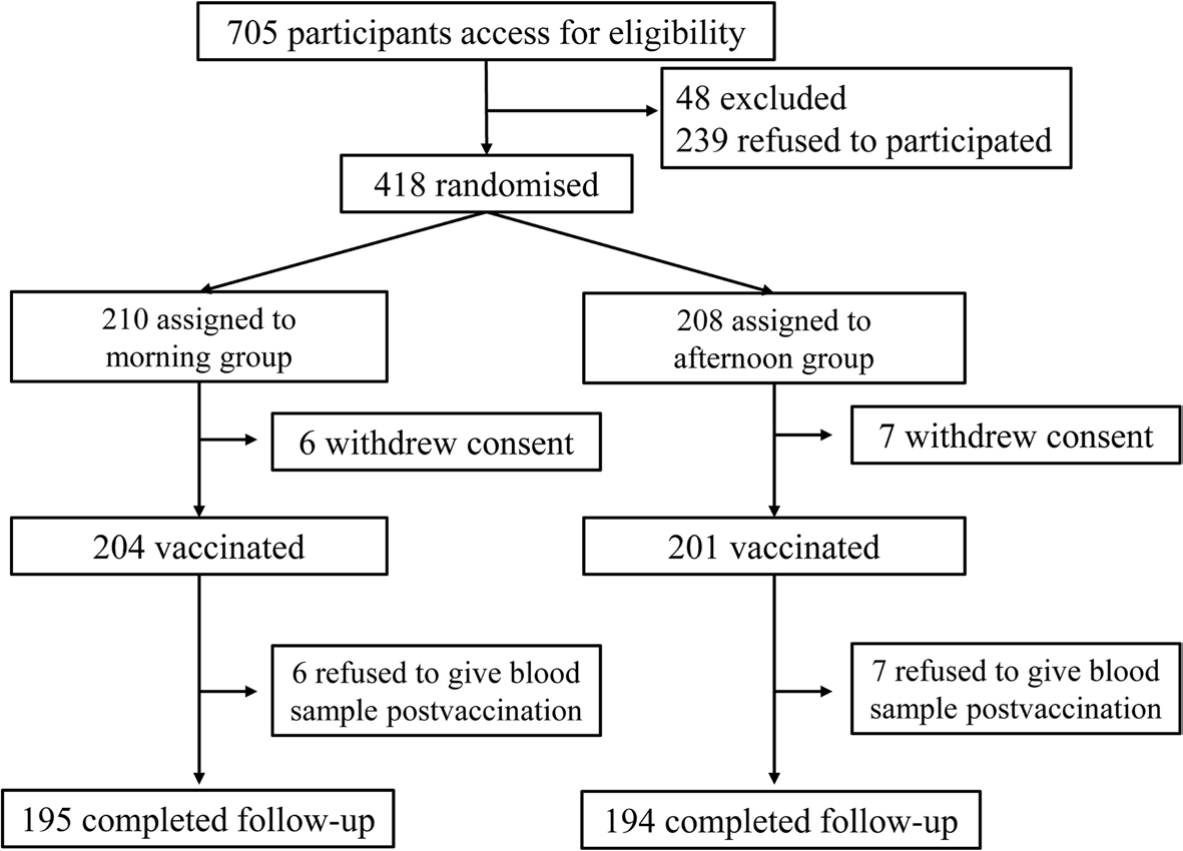
Flow diagram of the study

In total, 418 participants were enrolled between 27^th^ October 2020 and 22^nd^ December 2020 and provided blood samples before vaccination. Of these, 210 participants were randomized to morning and 208 to afternoon vaccination. One month after vaccination, 389 participants (195 in the morning group and 194 in the afternoon group) provided blood samples and completed the follow-up process (Fig. 1). Table 1 shows baseline characteristics of the morning and afternoon groups, which were largely similar across the two groups.

**Table 1:** Baseline characteristics of the morning group and the afternoon group

	Mean (SD)/ N (%)			
	Total	Morning	Afternoon	*P*
Gender				
Male	146 (37.5)	73 (37.4)	73 (37.6)	1.000
Female	243 (62.5)	122 (62.6)	121 (62.4)	
Age	62.8 (7.2)	62.9 (7.4)	62.7 (6.9)	0.668
50–60 years old	191 (49.1)	97 (49.7)	94 (48.5)	0.839
65–75 years old	198 (50.9)	98 (50.3)	100 (51.6)	
Smoking				
No	334 (85.9)	168 (86.2)	166 (85.6)	0.972
Past smoker	14 (3.6)	7 (3.6)	7 (3.6)	
Current smoker	41 (10.5)	20 (10.3)	21 (10.8)	
Alcohol consumption				
No	337 (86.6)	171 (87.7)	166 (85.6)	0.555
Yes	52 (13.4)	24 (12.3)	28 (14.4)	
Hypertension				
No	244 (62.7)	117 (60.0)	127 (65.5)	0.295
Yes	145 (37.3)	78 (40.0)	67 (34.5)	
Diabetes				
No	335 (86.1)	161 (82.6)	174 (89.7)	0.056
Yes	54 (13.9)	34 (17.4)	20 (10.3)	
Coronary heart disease				
No	356 (91.5)	179 (91.8)	177 (91.2)	0.858
Yes	33 (8.5)	16 (8.2)	17 (8.8)	
History of Influenza vaccination				
No	319 (82.0)	168 (86.2)	151 (77.8)	0.035
Yes	70 (18.0)	27 (13.9)	43 (22.2)	
Community distribution				
Shipai Street	193 (49.6)	100 (51.3)	93 (47.9)	0.543
Baiyun Street	196 (50.4)	95 (48.7)	101 (52.1)	
BMI	23.8 (3.0)	24.1 (3.0)	23.5 (3.0)	0.112
Sleep duration(h)	7.0 (1.4)	7.0 (1.4)	7.1 (1.3)	0.617
EQ-5D score	1.0 (0.0)	1.0 (0.0)	1.0 (0.0)	0.444

*Abbreviation*: *SD* Standard deviation, *BMI* Body Mass Index

### No significant difference between post-vaccination antibody titers in the morning compared to afternoon groups

**Fig. 2:**
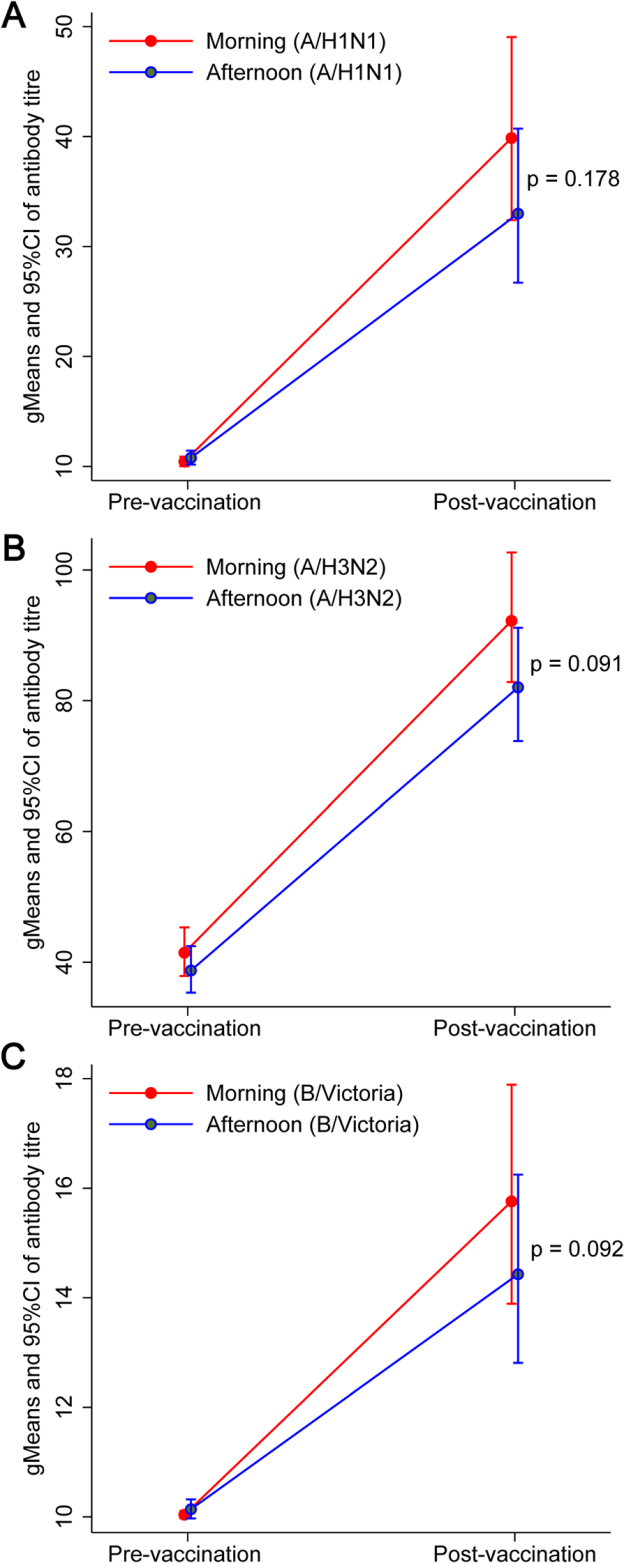
Antibody titers pre-vaccination and post-vaccination. **A** The overall geometric mean (95% CI) for antibody titers of A/H1N1 strain. **B** The overall geometric mean (95% CI) for antibody titers of A/H3N2 strain. **C** The overall geometric mean (95% CI) for antibody titers of B/Victoria strain

Figure 2 and Supplementary Table 1 show the data for antibody titers at baseline and follow-up for the whole study group. The morning and afternoon vaccination groups were similar in terms of their baseline antibody titers against H1N1, H3N2 and B strain antigens (all *p* > 0.05). There was a significant increase in antibody levels of all three strains one month after vaccination in both morning and afternoon groups (all *p* < 0.05).

Overall, there was no significant difference between post-vaccination antibody titers in the morning compared to afternoon groups: (gMean /95% CI) for A/H1N1 strain (39.9 (32.4, 49.1) vs. 33.0 (26.7, 40.7), *p* = 0.178); A/H3N2 strain (92.2 (82.8, 102.7) vs. 82.0 (73.8, 91.2), *p* = 0.091); B strain (15.8 (13.9, 17.9) vs. 14.4 (12.8, 16.3), *p* = 0.092). However, the seroconversion rate (HI antibody titers ≥ 10) of A/H1N1 of the morning group was higher than that of afternoon group (105 (53.85%) vs. 84 (43.30%), *p *= 0.043). There was no significant difference between the number of seroprotected individuals (HI antibody titers ≥ 40), the number of seroconverted individuals and fold change between post- and pre-vaccination gMean titer in the morning compared to afternoon groups for other strains (Supplementary Tables 1 and 2).

Adjusting for baseline titers, age and sex no significant association between one month antibody titers and vaccination time was found either (Supplementary Table 3).

### Morning vaccination enhances antibody responses in adults aged over 65

**Fig. 3:**
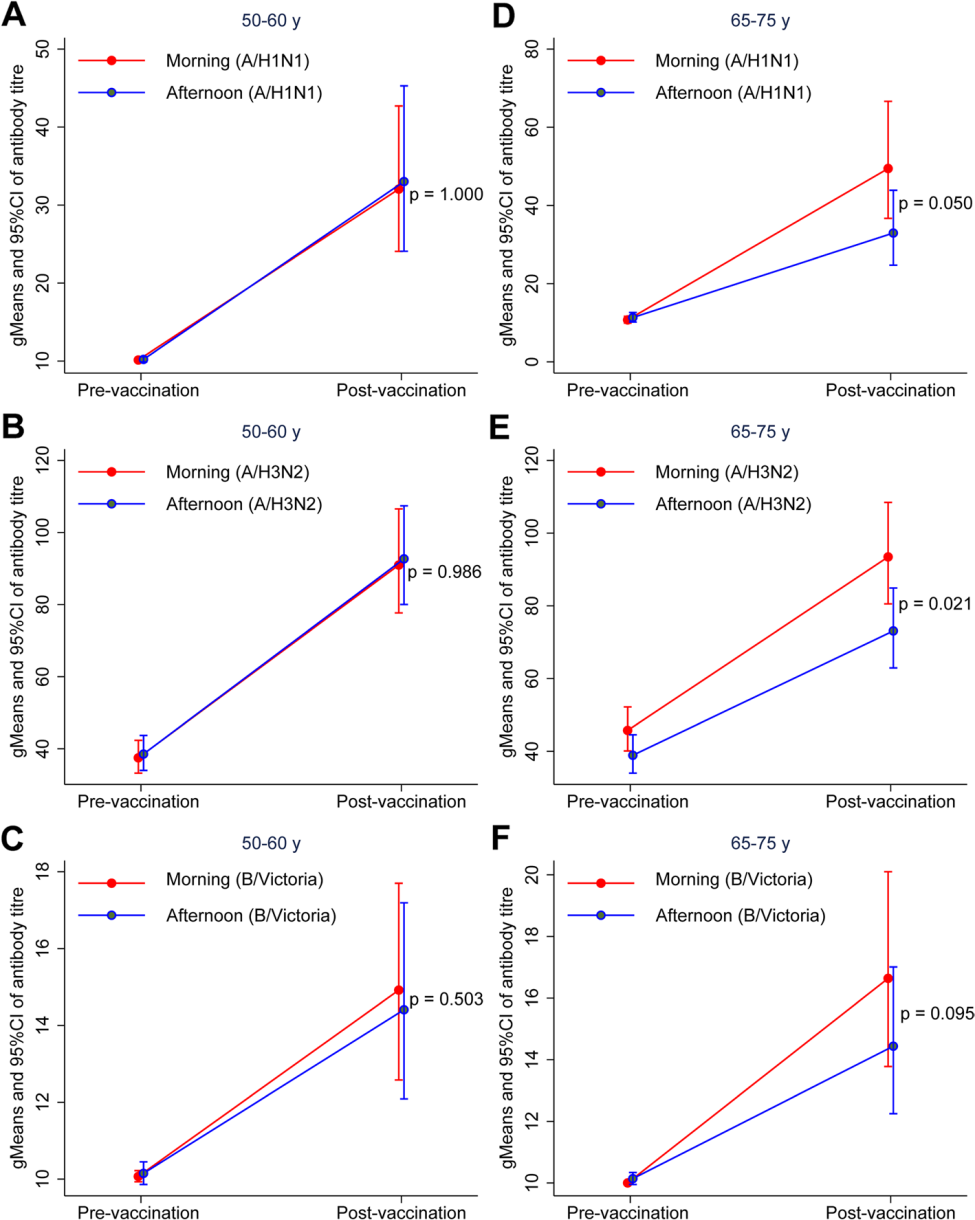
Antibody titers pre-vaccination and post-vaccination for the two age groups. **A**-**C** The geometric mean (95% CI) for antibody titers of A/H1N1, A/H3N2 and B/Victoria strain for aged 50–60 years. **D**-**F** The geometric mean (95% CI) for antibody titers of A/H1N1, A/H3N2 and B/Victoria strain for aged 65–75 years

Baseline antibody characteristics of the participants for the two age groups are shown in Supplementary Tables 4 and 5. In the subgroup aged 50–60 years, there were no significant differences between the antibody titers after vaccination for the morning and afternoon groups for the A/H1N1 strain, the A/H3N2 strain and the B strain (Fig. 3A-C and Supplementary Table 6). However, in the 65–75 years subgroup, morning vaccination resulted in a greater antibody response against the two A strains of influenza. Antibody titers for morning vs afternoon groups (gMean/95% CI) after vaccination were: (49.5 (36.7, 66.6) vs. 32.9 (24.7, 43.9), *p* = 0.050) for A/H1N1 strain; (93.5 (80.6, 108.5) vs. 73.1 (62.9, 84.9), *p* = 0.021) for the A/H3N2 strain and (16.6 (13.8, 20.1) vs. 14.4 (12.3, 17.0), *p* = 0.095) for the B strain, respectively (Fig. 3D-F and Supplementary Table 7).

In the 65–75 years subgroup, the seroconversion rate of A/H1N1 of the morning group was higher than that of the afternoon group (61 (62.24%) vs. 46 (46.00%), p = 0.023). The fold change of pre- and post-vaccination gMean titer (95%CI) in the morning was higher compared to afternoon groups (4.61 (3.42, 6.21)vs. 2.91 (2.18, 3.87), *p* = 0.023) (Supplementary Table 6– 9). There was a trend towards an interactive effect between age and vaccination time for the post-vaccination antibody titer of A/H1N1 strain (*p* = 0.058), but no significant interactive effect for A/H3N2 (*p *= 0.107) and B strains (*p* = 0.443) (Supplementary Table 10).

### Morning vaccination enhances antibody response in women but not men

**Fig. 4:**
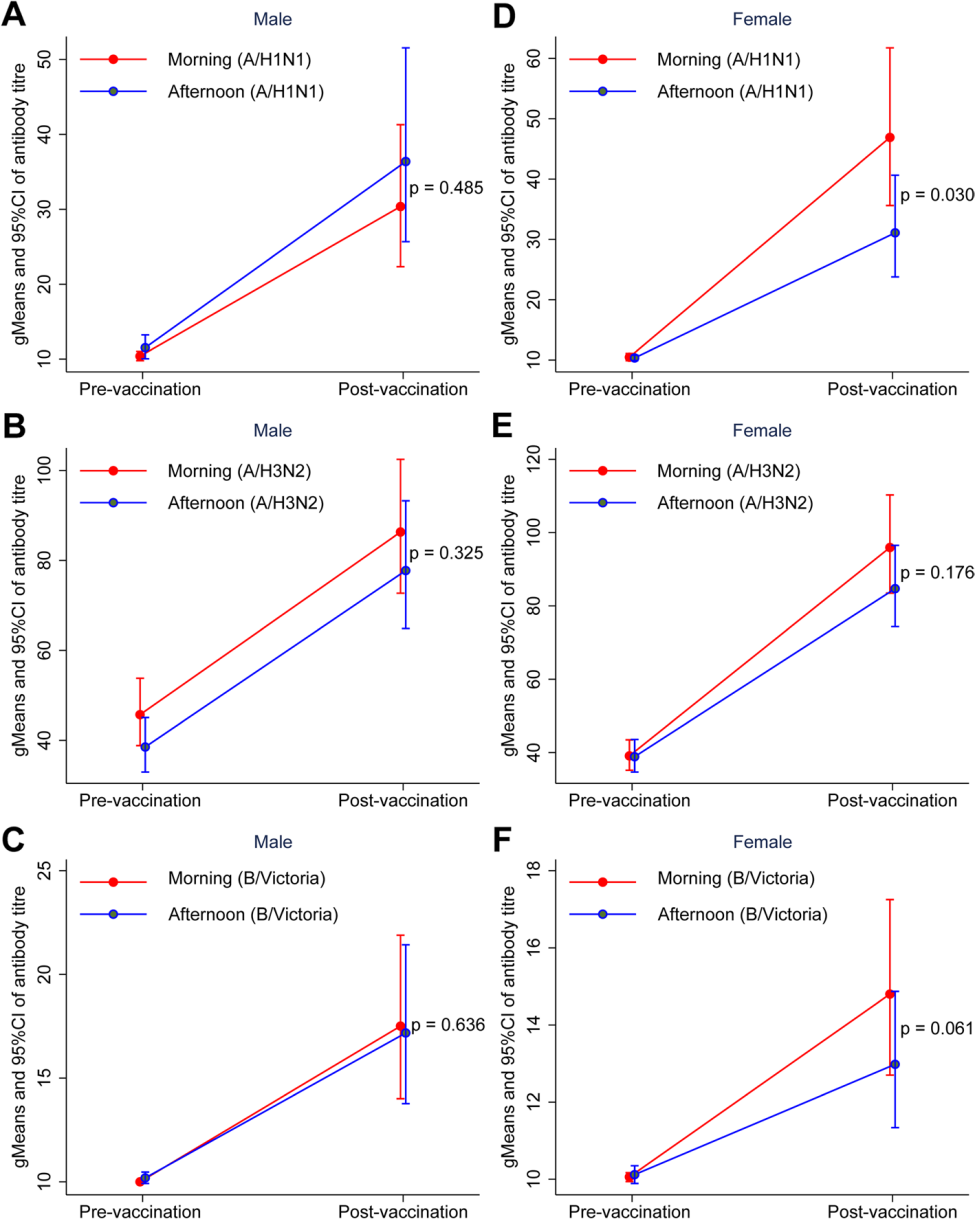
Antibody titers pre-vaccination and post-vaccination for subgroup analysis by gender. **A**-**C** The geometric mean (95% CI) for antibody titers of A/H1N1, A/H3N2 and B/Victoria strain for males. **D**-**F** The geometric mean (95% CI) for antibody titers of A/H1N1, A/H3N2 and B/Victoria strain for females

Baseline antibody characteristics of the participants of both sexes are shown in Supplementary Tables 11 and 12. Among males, there were no significant differences between the antibody titers after vaccination for morning and afternoon groups for the A/H1N1 strain, the A/H3N2 strain or the B strain (Fig. 4A-C and Supplementary Table 13). Among females, morning vaccination resulted in a greater antibody response to the A/H1N1 strain and was close to significance for the B strain. The antibody titers (gMean/95% CI) after morning and afternoon vaccination were (46.9 (35.6, 61.8) vs. 31.1 (23.8, 40.7), *p* = 0.03) for A/H1N1 strain, (96.0 (83.5, 110.3) vs. 84.7 (74.4, 96.5), *p* = 0.176) for the A/H3N2 strain and (14.8 (12.7, 17.3) vs. 13.0 (11.3, 14.9), *p* = 0.061) for the B strain, respectively (Fig. 4D-F and Supplementary Table 14).

In the female subgroup, the seroprotected and seroconverted rate of A/H1N1 of the morning group was higher than that of the afternoon group (64 (52.46%) vs. 37 (38.84%), *p* = 0.039), (67 (54.92%) vs. 49 (40.50%), *p* = 0.029), respectively. The fold change of pre- and post-vaccination gMean titer (95%CI) in the morning was higher compared to afternoon groups (4.48 (3.40, 5.90) vs.3.00 (2.29, 3.94), *p* = 0.036) (Supplementary Tables 13– 16).

There was a significant interactive effect between sex and vaccination time for the post-vaccination antibody titer of A/H1N1 strain (*p* = 0.043), but not for A/H3N2 (*p* = 0.553) or B/Victoria strain (*p* = 0.508) (Supplementary Table 10).

### Morning vaccination enhances antibody response in women aged over 65 only

We did a further 4-way comparison including men < 65 years, men > 65 years, women < 65 years and women > 65 years. The geometric mean for antibody titers of A/H1N1, and B/Victoria strain for women over 65 years in the morning group was significantly higher than that in the afternoon group (*p* = 0.006 and 0.014, respectively), but not for men and younger women (Supplementary Figs. 1 and 2).

### No significant differences between the occurrence of adverse events and clinical outcomes in the two groups

There were no significant differences between the occurrence of adverse events in the two groups. In the morning group, 14 (6.9%) participants had an adverse reaction, compared with 13 (6.5%) participants in the afternoon group (*p* = 1.000) (Supplementary Table 15). The most common adverse events in both groups were fatigue (3, 1.5% vs. 4, 2.0%). No serious adverse events were reported. No laboratory confirmed influenza and influenza-like symptoms were observed during the whole follow-up.

## Discussion

This study is the first individual-based randomized controlled trial to investigate the effect of timing of vaccination on the immune response to influenza vaccination. Overall, there was no significant difference between antibody titers post-vaccination in morning and afternoon groups but pre-specified subgroup analyses show that morning vaccination enhanced the immune response to the influenza vaccine in the older adults (65–75 years) and in women. Other studies also support a circadian response to vaccination. A recent study has shown that morning vaccination of Bacille Calmette-Guerin (BCG) vaccine elicits both a stronger trained immunity and adaptive immune response when compared to evening vaccination [12]. We also observed that morning vaccination showed a stronger antibody response to an inactivated vaccine against severe acute respiratory syndrome coronavirus 2 (SARS-CoV-2) in a non-randomised cohort study [13].

Older individuals are more prone to mortality and morbidity from influenza infections. This is exacerbated by their suboptimal immune response to vaccines [14], due to the age-related remodeling of the immune system (immune senescence) which includes thymic atrophy and a reduced production of naïve T cells [15]. This compromises immune responses to new pathogens and vaccines. This immune senescence may explain our observation that the time of day effect of vaccination was only seen in the older age group. In a system that is operating close to optimally, as in the younger age group, it is possible that the influence of circadian rhythms is not revealed.

There are many processes potentially underlying the effect of the time of day on vaccination responses. Several aspects of the immune system are influenced by circadian rhythms and the immune response varies at different times of day [16]. One possible explanation for a greater immune response to vaccination in the morning is the circadian profile of hormones such as cortisol and pro-inflammatory cytokines such as TNFα, both key modulators of immune responses [17, 18]. However, this is a less likely factor as cortisol, which is a potent immune suppressor, peaks in the morning when the vaccination response was higher. In addition, de Bree et al. showed that incubation of monocytes with serum taken from volunteers at either 8am or 6 pm did not induce differences in induction of trained immunity to BCG, suggesting that circadian immune responses are not primarily related to soluble factors [12].

That the circadian nature of the vaccine response may be based largely within immune cells is supported by several observations. Immune cells express clock genes which entrain a circadian rhythm in a wide variety of immune cell functions including cytokine and chemokine secretion, migration and the proliferative response to antigens [19]. For example, IL1-β is under the control of the clock gene BMAL1 [20], effecting a circadian response to immune challenge. In adaptive immunity, circadian control of T cell and B cell responses affects the amplitude and rhythmic proliferation responses of these cells by modulating the timing of T cell interactions with antigen-presenting cells [21]. It has also been shown that the frequency of different immune cells in the circulation varies with time of day, with CD4 T cells rising through the day from a low point in the morning [22]. Of relevance to the initial response to vaccination, it has been shown in mice that trafficking of monocytes from the blood to an inflamed site is subject to circadian variation regulated by BMAL1 expression in monocytes [23]. As well as modulation of the response to infection this could affect the response to new antigens including vaccines.

Here we also report an effect of sex on the circadian differences in vaccination responses, with only females showing the higher antibody titers in the morning. The literature in this area is minimal and contradictory. In a small observational study, only men vaccinated in the morning had a greater antibody response to the A/Panama/2007/99 influenza strain and the Hepatitis A vaccine [10], in contrast there was no interaction between time of vaccination and sex in our previous cluster trial on the influenza vaccine [11]. In the study reported here we also evaluated differences based on sex at different ages and found the geometric mean for antibody titers of A/H1N1, and B/Victoria strain for women over 65 years in the morning group was significantly higher than that in the afternoon group, but not for men and younger women. Although the influence of sex on circadian aspects of the vaccination response clearly needs further research, the effect of sex on vaccination responses is well established. Females produce higher antibody titers than males to a wide range of vaccines [24, 25], they also have higher B cell numbers [26] and this difference persists in to old age, it is thus surprising that it was the females that benefitted from the circadian effect rather than the men. Mechanisms involved in these sex differences are complex and include immunological, hormonal, behavioral, and genetic factors [27]. Research has shown that sex hormones may be key immune modulators, with vaccine-induced antibody responses increased in females by estradiol and decreased in males by testosterone [28]. As our older females would all be post-menopausal this may have allowed the circadian benefit to be revealed. For genetic influences, these may lie in genes carried on the x chromosome, including those encoding the pathogen recognition receptors TLR7 and TLR9 and important transcription factors such as FoxP3 [29].

One aspect of our data that was also seen in our previous study [11], was that the circadian effect on the influenza vaccine response was not seen to all strains in the vaccine. The immune response does vary significantly to components within multi-strain vaccines such as the influenza vaccine. The strain specific effects we observed may reflect the fact that the response to those strains was optimal, possibly due to prior exposure to the strain in our older subject group, and thus the circadian influence was minimized. Further studies are needed to explore the impact of circadian rhythms on the immune response to influenza vaccination of different strains.

Our study does have some limitations. One is that the sample size was insufficient to observe significant differences in clinical outcomes and in future a larger study will be required to determine if vaccine clinical efficacy also shows circadian effects. Secondly, cytokine profiles and influenza-specific T-cell and B-cells were not analysed in the current study, these data would help to provide improved understanding of the mechanisms involved in the time of day effect. Analysis of clock gene expression in key immune cells such as dendritic cells, T cells and B cells at the two time points used in our study would also provide mechanistic insight. A third limitation is that all baseline sera were collected in the morning to allow for randomization, but as half the group were then not vaccinated until the afternoon this may have masked a slight difference in baseline antibody titers which we reported previously [11].

## Conclusions

In summary, the data suggest that morning vaccination may result in stronger immunogenicity to inactivated influenza vaccines among older individuals and specifically in women. A large randomized controlled trial with clinical outcomes including laboratory confirmed infections or hospitalizations would be useful. Many routine national vaccination programs should lend themselves readily to such a trial. On the other hand, one could argue that since modifying vaccination programs to vaccinate older individuals in the morning is simple, eminently feasible in most systems, cost free, and unlikely to cause harm, the potential health gains from the moderate increase in immunogenicity would justify change of practice without a further large trial. Such a practice can, for example, be implemented readily in care homes. A large trial testing this question on the response of older individuals to COVID-19 vaccines or other vaccines may also be worthwhile.

## Methods

### Study design and participants

This randomized controlled trial was conducted in two community health service centers in Guangzhou, Guangdong, China: Shipai Street Community Health Service Center and Baiyun Street Community Health Service Center. Inclusion criteria were: (1) residents in Guangzhou, who lived in Guangzhou in the past six months; (2) people aged 50–60 or 65–75 years old. Exclusion criteria were: (1) autoimmune disease; (2) immunodeficiency syndrome; (3) malignant tumor; (4) taking drugs that may affect the immune function, such as immunosuppressant agents or immunopotentiators or glucocorticoids within one month before enrolment; (5) allergic to any of the ingredients in the vaccine.

This study was approved by the Ethics Committee of the First Affiliated Hospital, Sun Yat-Sen University. Written informed consent was obtained from each participant before enrolment. This study was registered in Chinese Clinical Trial Registry (NO. ChiCTR2000039568).

### Procedures

Participants were recruited through on-site approach at the two centers and online between October 27 and December 22, 2020. Eligible participants were randomly assigned to the morning (“intervention”) group and the afternoon (“control”) group. Blood samples were collected from all the participants before vaccination in the morning. Participants in the intervention group were vaccinated in the morning (9-11am), while participants in the control group were vaccinated in the afternoon (3-5 pm) by the nurses in the centers. All participants were asked to attend on-site follow-up one month after vaccination and follow-up via telephone three months after vaccination.

### Interventions

We used a trivalent inactivated influenza vaccine (Sanofi Pasteur split virion, 2020/2021 strains), which contained three viral strains: 15 µg of A/ Guangdong-Maonan/ SWL1536/2019 (H1N1) pdm09-like virus, 15 µg of A/ HongKong/ 2671/2019 (H3N2)-like virus and 15 µg of B/ Washington/ 02/2019 (B/ Victoria lineage)-like virus. It was delivered as a single intramuscular injection in the deltoid muscle, using the standard single dose (0.5 ml). Participants were monitored for 30 min after injection for immediate adverse reactions.

### Data collection and follow-up

Before enrolment, all participants were asked to complete a screening questionnaire that included socio-demographics (age, gender, etc.), health behaviors (smoking, alcohol consumption, sleep duration and exercise time), medical history, allergic history, history of influenza vaccination, history of laboratory confirmed influenza and the EuroQol five dimension (EQ-5D) scale.

Participants were followed up for any reactions and adverse events within 28 days post vaccination. Serious adverse events self-reported by participants were documented throughout the study.

At the on-site follow-up one month after vaccination, all participants were asked to give a morning fasted blood sample and finish a brief questionnaire. The questionnaire was designed to collect the information about the occurrence of influenza-like symptoms, laboratory confirmed influenza, adverse events and EQ-5D scale. At the follow-up by telephone three months after vaccination, the participants were asked about the occurrence of influenza-like symptoms and laboratory confirmed influenza.

Blood samples were collected from all the participants at 8–10 am before vaccination and one month after vaccination, respectively. On each occasion, 5 ml of peripheral venous blood were collected into tubes containing inert separating gel and coagulant. Then blood samples were centrifuged at 3000 rpm for 10 min, and the separated serum was frozen at − 80 ◦C for later analysis.

### Haemagglutination inhibition (HAI) assay

The antibody titers against each of the three seasonal strains of influenza in the vaccine (H1N1 A, H3N2 A and B/Victoria strain) were measured using HAI assay [30]. In the original protocol first registered at Chinese Clinical Trial Registry, it was stated that antibody titers would be measured by enzyme linked immune sorbent assay (ELISA) (Jingmei, China) instead of HAI. However, we analysed a small number of samples (*n* = 9) as a pilot and found that the concentrations of IgG, IgA, IgM in the sera changed little after vaccination, suggesting a lack of specificity of the ELISA kits we used. We therefore changed the method of assay to HAI, which is also in line with the methods used in previous trials on influenza vaccines [31, 32]. This analysis was conducted following the protocol recommended by WHO and the Influenza Surveillance Network for the surveillance of influenza viruses and vaccine efficacy [33].

### Outcomes

The primary outcome of this study was antibody titer one-month post-vaccination. Secondary outcomes reported here were: (1) adverse events; (2) the occurrence of influenza-like symptoms (defined as an acute respiratory illness with a measured temperature of ≥ 38 °C and cough, with onset within the past 10 days) and laboratory confirmed influenza [34].

### Randomization and masking

The participants were randomly assigned to the intervention group and the control group at a 1:1 ratio via a computer-based randomization program based on stratified block randomization. The stratification factors were age (50–60 years old and 65–75 years old) and gender (male and female). Block size was randomly assigned as 4 or 6. Due to the nature of vaccination time the participants could not be blinded. The investigators responsible for outcome assessment and laboratory test were blinded to the allocation.

### Statistical analysis

The sample size in this study was calculated based on the level of antibody titer. According to our previous study [11], the mean (log10) difference of antibody titer of three virus strains between morning and afternoon group were 2.5, 1.7, and 1.2, respectively. The smallest mean (log10) difference of 1.2 with the standard deviation of 3.5 were used to the sample size. The two-sided type I error rate was 5% and the type II error rate was 10%. Considering 10% loss of follow-up or non-compliance, the sample size was determined as 200 cases per group.

The primary analysis population (per-protocol population) included all study participants who underwent the vaccination, and whose pre-vaccination and post-vaccination antibody titers were available.

Antibody titers were described as mean (standard deviation, SD), median (interquartile range, IQR) and geometric mean (gMean) (95% confidence interval, CI). The seroconverted individuals were defined as those with HI antibody titers ≥ 10, while the seroprotected individuals were defined as those with HI antibody titers ≥ 40. The fold change in the antibody titers after the vaccination were calculated using post-vaccination divided by pre-vaccination antibody titers. The change of antibody titer level between baseline and one month later was examined by paired Wilcoxon signed rank test. The comparisons of antibody titers between the two groups used Mann–Whitney U test. Analyses for safety and clinical outcomes included participants who underwent the vaccination and provided the information on safety and clinical outcome. Adverse events were described as the number of cases (percentage) and compared by Fisher’s exact test between the two groups. Pre-specified subgroup analyses were performed according to the stratification factors, age (50–60 years, 65–75 years) and gender (male, female). Linear regressions were used to analyze the effect of vaccination at different time of day on one month antibody titer adjusted baseline antibody titer and stratification factors. Log-transformed titer was used in linear regression analysis. To determine whether any intervention effects were mediated by age or gender, the interaction terms of vaccination time × age and vaccination time × gender were entered in the models.

For socio-demographic characteristics, continuous variables were described as mean (SD) or median (IQR), and the categorical variables were described as the number of cases (percentage). EQ-5D scores were calculated based on the nationally representative Chinese Time Trade-Off Value Set for EQ-5D-3L health states, where 1 represented ‘full health’ and values lower than 1 represented ‘worse health’ [35].

## Supplementary Information

**Additional file 1. **

## Data Availability

The datasets used and/or analysed during the current study are available from the corresponding author on reasonable request.

## Abbreviations

COVID-19: Corona Virus Disease 2019
BCG: Bacille Calmette-Guerin
SARS-CoV-2: Severe Acute Respiratory Syndrome Coronavirus 2
EQ-5D: EuroQol Five Dimension
HAI: Haemagglutination inhibition
ELISA: Enzyme Linked Immune Sorbent Assay
SD: Standard Deviation
IQR: Interquartile Range
CI: Confidence Interval
gMean: Geometric Mean
