# Supplementary material for: The impact of circadian rhythms on the immune response to influenza vaccination in middle-aged and older adults (IMPROVE): a randomised controlled trial
Source: Immun Ageing. 2022 Oct 17;19:46. doi: 10.1186/s12979-022-00304-w (PMC9574181; doi:10.1186/s12979-022-00304-w)
Supplement: Supplementary file 1 — Additional file 1. [file 12979_2022_304_MOESM1_ESM.docx]

**Supplementary Table 1 The antibody titers pre-vaccination and post-vaccination in morning and afternoon groups**

|  |  |  | Morning(N=195) | Afternoon(N=194) | *P* |
| --- | --- | --- | --- | --- | --- |
| Prevaccination | A/H1N1 | Mean (SD) | 11.6 (12.8) | 13.5 (25.8) | 0.390 |
|  |  | gMean (95%CI) | 10.4 (10.0, 10.9) | 10.8 (10.2, 11.4) |  |
|  |  | Median (IQR) | 10.0 (10.0, 10.0) | 10.0 (10.0, 10.0) |  |
|  | A/H3N2 | Mean (SD) | 50.5 (35.9) | 46.9 (29.3) | 0.418 |
|  |  | gMean (95%CI) | 41.5 (37.9, 45.3) | 38.7 (35.3, 42.5) |  |
|  |  | Median (IQR) | 40.0 (40.0, 80.0) | 40.0 (40.0, 80.0) |  |
|  | B/Victoria | Mean (SD) | 10.1 (0.7) | 10.3 (2.4) | 0.312 |
|  |  | gMean (95%CI) | 10.0 (10.0, 10.1) | 10.1 (10.0, 10.3) |  |
|  |  | Median (IQR) | 10.0 (10.0, 10.0) | 10.0 (10.0, 10.0) |  |
| Post-vaccination | A/H1N1 | Mean (SD) | 103.7 (123.5) | 95.9 (125.5) | 0.178 |
|  |  | gMean (95%CI) | 39.9 (32.4, 49.1) | 33.0 (26.7, 40.7) |  |
|  |  | Median (IQR) | 20.0 (10.0, 160.0) | 10.0 (10.0, 160.0) |  |
|  | A/H3N2 | Mean (SD) | 121.8 (94.3) | 108.6 (88.3) | 0.091 |
|  |  | gMean (95%CI) | 92.2 (82.8, 102.7) | 82.0 (73.8, 91.2) |  |
|  |  | Median (IQR) | 80.0 (80.0, 160.0) | 80.0 (40.0, 160.0) |  |
|  | B/Victoria | Mean (SD) | 29.7 (52.7) | 25.9 (46.2) | 0.092 |
|  |  | gMean (95%CI) | 15.8 (13.9, 17.9) | 14.4 (12.8, 16.3) |  |
|  |  | Median (IQR) | 10.0 (10.0, 20.0) | 10.0 (10.0, 10.0) |  |
| Fold change | A/H1N1 | Mean (SD) | 10.02 (12.20) | 9.07 (12.29) | 0.100 |
|  |  | gMean (95%CI) | 3.82(3.11, 4.69) | 3.06(2.48, 3.77) |  |
|  |  | Median (IQR) | 2.00(1.00, 16.00) | 1.00(1.00, 16.00) |  |
|  | A/H3N2 | Mean (SD) | 3.70 (5.34) | 3.23 (3.85) | 0.522 |
|  |  | gMean (95%CI) | 2.23(1.94, 2.55) | 2.12(1.87, 2.40) |  |
|  |  | Median (IQR) | 2.00(1.00, 4.00) | 2.00(1.00, 4.00) |  |
|  | B/Victoria | Mean (SD) | 2.97 (5.27) | 2.51 (4.51) | 0.089 |
|  |  | gMean (95%CI) | 1.57(1.38, 1.78) | 1.42(1.27, 1.60) |  |
|  |  | Median (IQR) | 1.00(1.00, 2.00) | 1.00(1.00, 1.00) |  |

Abbreviation: SD: standard deviation; IQR: interquartile range; gMean: geometric mean; CI: confidence interval.

**Supplementary Table 2 The overall number of seroprotected and seroconverted individuals pre- and post-vaccination**

|  | | Morning(N=195) | Afternoon(N=194) | P-values |
| --- | --- | --- | --- | --- |
| A/H1N1 seroprotected  N (%) | pre-vaccination | 3 (1.54) | 6 (3.09) | 0.337 |
|  | post-vaccination | 97 (49.74) | 80 (41.24) | 0.104 |
| A/H1N1 seroconverted  N (%) | pre-vaccination | 9 (4.62) | 12 (6.19) | 0.511 |
|  | post-vaccination | 105 (53.85) | 84 (43.30) | 0.043 |
| A/H3N2 seroprotected  N (%) | pre-vaccination | 149 (76.41) | 146 (75.26) | 0.814 |
|  | post-vaccination | 188 (96.41) | 186 (95.88) | 0.799 |
| A/H3N2 seroconverted  N (%) | pre-vaccination | 186 (95.38) | 178 (91.75) | 0.154 |
|  | post-vaccination | 193 (98.97) | 192 (98.97) | 1.000 |
| B/Victoria seroprotected  N (%) | pre-vaccination | 0 (0.00) | 1 (0.52) | 0.499 |
|  | post-vaccination | 35 (17.95) | 33 (17.01) | 0.894 |
| B/Victoria seroconverted  N (%) | pre-vaccination | 1 (0.51) | 4 (2.06) | 0.215 |
|  | post-vaccination | 60 (30.77) | 46 (23.71) | 0.139 |

**Supplementary Table 3 Multi-variable linear regression for post-vaccination antibody titer**

| Variables | Log - A/H1N1 | |  | Log - A/H3N2 | |  | Log - B | |
| --- | --- | --- | --- | --- | --- | --- | --- | --- |
|  | beta  (95% CI) | *P* |  | beta  (95% CI) | *P* |  | beta  (95% CI) | *P* |
| Log - baseline titer | 0.57  (0.16, 0.99) | 0.007 |  | 0.18  (0.06, 0.29) | 0.003 |  | 1.19  (0.26, 2.11) | 0.012 |
| Age (65-75 y) | 0.21  (-0.09, 0.50) | 0.175 |  | -0.11  (-0.26, 0.04) | 0.161 |  | 0.02  (-0.15, 0.20) | 0.797 |
| Gender (Female) | 0.21  (-0.10, 0.51) | 0.188 |  | 0.09  (-0.07, 0.24) | 0.262 |  | -0.22  (-0.40, -0.04) | 0.017 |
| Vaccination time (Morning) | 0.21  (-0.08, 0.50) | 0.158 |  | 0.10  (-0.05, 0.25) | 0.171 |  | 0.10  (-0.07, 0.27) | 0.243 |

Abbreviation: CI: confidence interval.

**Supplementary Table 4 Baseline characteristics of the morning and afternoon groups among participants aged 50-60 years old**

|  | Mean (SD)/ N (%) | | | | |
| --- | --- | --- | --- | --- | --- |
|  | Total | Morning | Afternoon | *P* | |
| N | 191 | 97 | 94 |  | |
| Gender | | | | | |
| Male | 55 (28.8) | 28 (28.9) | 27 (28.7) | 1.000 | |
| Female | 136 (71.2) | 69 (71.1) | 67 (71.3) |  |  |
| Age | 56.2 (3.2) | 56.3 (3.4) | 56.2 (2.9) | 0.991 | |
| Smoking | | | | | |
| No | 164 (85.9) | 82 (84.5) | 82 (87.2) | 0.468 | |
| Past smoker | 8 (4.2) | 3 (3.1) | 5 (5.3) |  |  |
| Current smoker | 19 (10.0) | 12 (12.4) | 7 (7.5) |  |  |
| Alcohol consumption | | | | | |
| No | 164 (85.9) | 84 (86.6) | 80 (85.1) | 0.837 | |
| Yes | 27 (14.1) | 13 (13.4) | 14 (14.9) |  |  |
| Hypertension | | | | |  |
| No | 141 (73.8) | 70 (72.2) | 71 (75.5) | 0.625 | |
| Yes | 50 (26.2) | 27 (27.8) | 23 (24.5) |  |  |
| Diabetes | | | | | |
| No | 174 (91.1) | 85 (87.6) | 89 (94.7) | 0.126 | |
| Yes | 17 (8.9) | 12 (12.4) | 5 (5.3) |  |  |
| Coronary heart disease | | | | | |
| No | 187 (97.9) | 95 (97.9) | 92 (97.9) | 1.000 | |
| Yes | 4 (2.1) | 2 (2.1) | 2 (2.1) |  |  |
| History of Influenza vaccination | | | | | |
| No | 170 (89.0) | 89 (91.8) | 81 (86.2) | 0.253 | |
| Yes | 21 (11.0) | 8 (8.3) | 13 (13.8) |  |  |
| Community distribution |  |  |  |  | |
| Shipai Street | 82 (42.9) | 43 (44.3) | 39 (41.5) | 0.770 | |
| Baiyun Street | 109 (57.1) | 54 (55.7) | 55 (58.5) |  |  |
| BMI | 23.6 (2.8) | 23.9 (2.8) | 23.3 (2.8) | 0.212 | |
| Sleep duration(h) | 7.2 (1.2) | 7.2 (1.3) | 7.2 (1.1) | 0.736 | |
| EQ-5D score | 1.0 (0.0) | 1.0 (0.0) | 1.0 (0.0) | 0.374 | |

Abbreviation: SD: standard deviation; BMI: Body Mass Index.

**Supplementary Table 5 Baseline characteristics of the morning and afternoon groups among participants aged 65-75 years old**

|  | Mean (SD)/ N (%) | | | | |
| --- | --- | --- | --- | --- | --- |
|  | Total | Morning | Afternoon | *P* | |
| N | 198 | 98 | 100 |  | |
| Gender | | | | | |
| Male | 91 (46.0) | 45 (45.9) | 46 (46.0) | 1.000 | |
| Female | 107 (54.0) | 53 (54.1) | 54 (54.0) |  |  |
| Age | 69.2 (2.9) | 69.6 (3.1) | 68.8 (2.7) | 0.110 | |
| Smoking | | | | | |
| No | 170 (85.9) | 86 (87.8) | 84 (84.0) | 0.369 | |
| Past smoker | 6 (3.0) | 4 (4.1) | 2 (2.0) |  |  |
| Current smoker | 22 (11.1) | 8 (8.2) | 14 (14.0) |  |  |
| Alcohol consumption | | | | | |
| No | 173 (87.4) | 87 (88.8) | 86 (86.0) | 0.670 | |
| Yes | 25 (12.6) | 11 (11.2) | 14 (14.0) |  |  |
| Hypertension | | | | |  |
| No | 103 (52.0) | 47 (48.0) | 56 (56.0) | 0.319 | |
| Yes | 95 (48.0) | 51 (52.0) | 44 (44.0) |  |  |
| Diabetes | | | | | |
| No | 161 (81.3) | 76 (77.6) | 85 (85.0) | 0.204 | |
| Yes | 37 (18.7) | 22 (22.5) | 15 (15.0) |  |  |
| Coronary heart disease | | | | | |
| No | 169 (85.4) | 84 (85.7) | 85 (85.0) | 1.000 | |
| Yes | 29 (14.7) | 14 (14.3) | 15 (15.0) |  |  |
| History of Influenza vaccination | | | | | |
| No | 149 (75.3) | 79 (80.6) | 70 (70.0) | 0.100 | |
| Yes | 49 (24.8) | 19 (19.4) | 30 (30.0) |  |  |
| Community distribution |  |  |  |  | |
| Shipai Street | 111 (56.1) | 57 (58.2) | 54 (54.0) | 0.570 | |
| Baiyun Street | 87 (43.9) | 41 (41.8) | 46 (46.0) |  |  |
| BMI | 24.0 (3.2) | 24.4 (3.2) | 23.6 (3.2) | 0.311 | |
| Sleep duration(h) | 6.8 (1.5) | 6.7 (1.5) | 7.0 (1.4) | 0.413 | |
| EQ-5D score | 1.0 (0.0) | 1.0 (0.0) | 1.0 (0.0) | 0.824 | |

Abbreviation: SD: standard deviation; BMI: Body Mass Index.

**Supplementary Table 6 The antibody titers prevaccination and post-vaccination of adults aged 50-60**

|  |  |  | Morning（N=97） | Afternoon（N=94） | *P* |
| --- | --- | --- | --- | --- | --- |
| Prevaccination | A/H1N1 | Mean (SD) | 10.2 (1.4) | 10.4 (3.3) | 0.964 |
|  |  | gMean (95%CI) | 10.1 (9.9, 10.4) | 10.2 (9.9, 10.6) |  |
|  |  | Median (IQR) | 10.0 (10.0, 10.0) | 10.0 (10.0, 10.0) |  |
|  | A/H3N2 | Mean (SD) | 44.1 (23.8) | 45.5 (25.6) | 0.697 |
|  |  | gMean (95%CI) | 37.5 (33.2, 42.3) | 38.6 (34.0, 43.7) |  |
|  |  | Median (IQR) | 40.0 (20.0, 80.0) | 40.0 (40.0, 80.0) |  |
|  | B/Victoria | Mean (SD) | 10.1 (1.0) | 10.3 (3.1) | 0.971 |
|  |  | gMean (95%CI) | 10.1 (9.9, 10.2) | 10.2 (9.9, 10.5) |  |
|  |  | Median (IQR) | 10.0 (10.0, 10.0) | 10.0 (10.0, 10.0) |  |
| Post-vaccination | A/H1N1 | Mean (SD) | 86.4 (114.9) | 101.4 (131.5) | 1.000 |
|  |  | gMean (95%CI) | 32.1 (24.1, 42.7) | 33.0 (24.1, 45.3) |  |
|  |  | Median (IQR) | 10.0 (10.0, 160.0) | 10.0 (10.0, 160.0) |  |
|  | A/H3N2 | Mean (SD) | 121.1 (94.8) | 120.1 (93.0) | 0.986 |
|  |  | gMean (95%CI) | 91.0 (77.7, 106.5) | 92.7 (80.0, 107.4) |  |
|  |  | Median (IQR) | 80.0 (80.0, 160.0) | 80.0 (80.0, 160.0) |  |
|  | B/Victoria | Mean (SD) | 26.0 (40.6) | 26.0 (42.0) | 0.503 |
|  |  | gMean (95%CI) | 14.9 (12.6, 17.7) | 14.4 (12.1, 17.2) |  |
|  |  | Median (IQR) | 10.0 (10.0, 10.0) | 10.0 (10.0, 10.0) |  |
| Fold change | A/H1N1 | Mean (SD) | 8.45 (11.28) | 9.86 (12.97) | 0.973 |
|  |  | gMean (95%CI) | 3.16(2.38, 4.20) | 3.23(2.36, 4.42) |  |
|  |  | Median (IQR) | 1.00(1.00, 16.00) | 1.00(1.00, 16.00) |  |
|  | A/H3N2 | Mean (SD) | 4.04 (5.76) | 3.18 (2.77) | 0.993 |
|  |  | gMean (95%CI) | 2.43(2.00, 2.95) | 2.40(2.07, 2.80) |  |
|  |  | Median (IQR) | 2.00(1.00, 4.00) | 2.00(2.00, 4.00) |  |
|  | B/Victoria | Mean (SD) | 2.59 (4.06) | 2.47 (3.96) | 0.578 |
|  |  | gMean (95%CI) | 1.48(1.25, 1.76) | 1.42(1.20, 1.68) |  |
|  |  | Median (IQR) | 1.00(1.00, 1.00) | 1.00(1.00, 1.00) |  |

Abbreviation: SD: standard deviation; IQR: interquartile range; gMean: geometric mean; CI: confidence interval.

**Supplementary Table 7 The antibody titers prevaccination and post-vaccination of adults aged 65-75**

|  |  |  | Morning（N=98） | Afternoon（N=100） | *P* |
| --- | --- | --- | --- | --- | --- |
| Prevaccination | A/H1N1 | Mean (SD) | 13.0 (18.0) | 16.4 (35.6) | 0.327 |
|  |  | gMean (95%CI) | 10.7 (9.9, 11.6) | 11.3 (10.2, 12.6) |  |
|  |  | Median (IQR) | 10.0 (10.0, 10.0) | 10.0 (10.0, 10.0) |  |
|  | A/H3N2 | Mean (SD) | 56.8 (44.0) | 48.2 (32.5) | 0.134 |
|  |  | gMean (95%CI) | 45.8 (40.1, 52.2) | 38.9 (34.0, 44.5) |  |
|  |  | Median (IQR) | 40.0 (40.0, 80.0) | 40.0 (20.0, 80.0) |  |
|  | B/Victoria | Mean (SD) | 10.0 (0.0) | 10.2 (1.4) | 0.160 |
|  |  | gMean (95%CI) | 10.0 (10.0, 10.0) | 10.1 (10.0, 10.3) |  |
|  |  | Median (IQR) | 10.0 (10.0, 10.0) | 10.0 (10.0, 10.0) |  |
| Post-vaccination | A/H1N1 | Mean (SD) | 120.8 (129.8) | 90.7 (120.1) | 0.050 |
|  |  | gMean (95%CI) | 49.5 (36.7, 66.6) | 32.9 (24.7, 43.9) |  |
|  |  | Median (IQR) | 40.0 (10.0, 320.0) | 10.0 (10.0, 160.0) |  |
|  | A/H3N2 | Mean (SD) | 122.5 (94.3) | 97.7 (82.6) | 0.021 |
|  |  | gMean (95%CI) | 93.5 (80.6, 108.5) | 73.1 (62.9, 84.9) |  |
|  |  | Median (IQR) | 80.0 (80.0, 160.0) | 80.0 (40.0, 160.0) |  |
|  | B/Victoria | Mean (SD) | 33.5 (62.3) | 25.9 (50.1) | 0.095 |
|  |  | gMean (95%CI) | 16.6 (13.8, 20.1) | 14.4 (12.3, 17.0) |  |
|  |  | Median (IQR) | 10.0 (10.0, 20.0) | 10.0 (10.0, 10.0) |  |
| Fold change | A/H1N1 | Mean (SD) | 11.57 (12.93) | 8.32 (11.64) | 0.023 |
|  |  | gMean (95%CI) | 4.61(3.42, 6.21) | 2.91(2.18, 3.87) |  |
|  |  | Median (IQR) | 4.00(1.00, 32.00) | 1.00(1.00, 16.00) |  |
|  | A/H3N2 | Mean (SD) | 3.36 (4.90) | 3.29 (4.65) | 0.331 |
|  |  | gMean (95%CI) | 2.04(1.69, 2.46) | 1.88(1.55, 2.28) |  |
|  |  | Median (IQR) | 2.00(1.00, 4.00) | 2.00(1.00, 4.00) |  |
|  | B/Victoria | Mean (SD) | 3.35 (6.23) | 2.54 (4.99) | 0.075 |
|  |  | gMean (95%CI) | 1.66(1.38, 2.01) | 1.42(1.21, 1.68) |  |
|  |  | Median (IQR) | 1.00(1.00, 2.00) | 1.00(1.00, 1.00) |  |

Abbreviation: SD: standard deviation; IQR: interquartile range; gMean: geometric mean; CI: confidence interval

**Supplementary Table 8 The number of seroprotected and seroconverted individuals pre- and post-vaccination for adults aged over 65**

|  |  | Morning(N=98) | Afternoon(N=100) | P-values |
| --- | --- | --- | --- | --- |
| A/H1N1 seroprotected  N (%) | pre-vaccination | 3 (3.06) | 5 (5.00) | 0.721 |
|  | post-vaccination | 56 (57.14) | 43 (43.00) | 0.064 |
| A/H1N1 seroconverted  N (%) | pre-vaccination | 4 (4.08) | 7 (7.00) | 0.537 |
|  | post-vaccination | 61 (62.24) | 46 (46.00) | 0.023 |
| A/H3N2 seroprotected  N (%) | pre-vaccination | 81 (82.65) | 74 (74.00) | 0.169 |
|  | post-vaccination | 94 (95.92) | 93 (93.00) | 0.537 |
| A/H3N2 seroconverted  N (%) | pre-vaccination | 95 (96.94) | 91 (91.00) | 0.134 |
|  | post-vaccination | 98 (100.00) | 99 (99.00) | 1.000 |
| B/Victoria seroprotected  N (%) | pre-vaccination | 0 (0.00) | 0 (0.00) | / |
|  | post-vaccination | 18 (18.37) | 18 (18.00) | 1.000 |
| B/Victoria seroconverted  N (%) | pre-vaccination | 0 (0.00) | 2 (2.00) | 0.498 |
|  | post-vaccination | 37 (37.76) | 25 (25.00) | 0.066 |

**Supplementary Table 9 The number of seroprotected and seroconverted individuals pre- and post-vaccination for adults aged 50-60**

|  |  | Morning(N=97) | Afternoon(N=94) | P-values |
| --- | --- | --- | --- | --- |
| A/H1N1 seroprotected N (%) | pre-vaccination | 0 (0.00) | 1 (1.06) | 0.492 |
|  | post-vaccination | 41 (42.27) | 37 (39.36) | 0.769 |
| A/H1N1 seroconverted N (%) | pre-vaccination | 5 (5.15) | 5 (5.32) | 1.000 |
|  | post-vaccination | 44 (45.36) | 38 (40.43) | 0.559 |
| A/H3N2 seroprotected N (%) | pre-vaccination | 68 (70.10) | 72 (76.60) | 0.331 |
|  | post-vaccination | 94 (96.91) | 93 (98.94) | 0.621 |
| A/H3N2 seroconverted N (%) | pre-vaccination | 91 (93.81) | 87 (92.55) | 0.780 |
|  | post-vaccination | 95 (97.94) | 93 (98.94) | 1.000 |
| B/Victoria seroprotected N (%) | pre-vaccination | 0 (0.00) | 1 (1.06) | 0.492 |
|  | post-vaccination | 17 (17.53) | 15 (15.96) | 0.847 |
| B/Victoria seroconverted N (%) | pre-vaccination | 1 (1.03) | 2 (2.13) | 0.617 |
|  | post-vaccination | 23 (23.71) | 21 (22.34) | 0.865 |

**Supplementary Table 10 Multi-variable linear regression for one month antibody titer with the interactive terms**

| Variables | Log - A/H1N1 | |  | Log - A/H3N2 | |  | Log - B | |
| --- | --- | --- | --- | --- | --- | --- | --- | --- |
|  | beta  (95% CI) | *P* |  | beta  (95% CI) | *P* |  | beta  (95% CI) | *P* |
| Log - baseline titer | 0.55  (0.14, 0.97) | 0.009 |  | 0.17  (0.06, 0.29) | 0.004 |  | 1.18  (0.25, 2.11) | 0.013 |
| Age (65-75 y) | -0.08  (-0.50, 0.34) | 0.713 |  | -0.23  (0.45, -0.02) | 0.033 |  | -0.05  (-0.29, 0.20) | 0.717 |
| Gender (Female) | -0.11  (-0.54, 0.32) | 0.612 |  | 0.04  (-0.18, 0.26) | 0.711 |  | -0.28  (-0.53, -0.03) | 0.031 |
| Vaccination time (Morning) | -0.47  (-1.08, 0.13) | 0.121 |  | -0.08  (-0.39, 0.23) | 0.604 |  | -0.04  (-0.40, 0.31) | 0.811 |
| Morning * 65-75 | 0.57  (-0.02, 1.16) | 0.058 |  | 0.25  (-0.05, 0.55) | 0.107 |  | 0.14  (-0.21, 0.48) | 0.443 |
| Morning * Female | 0.63  (0.02, 1.24) | 0.043 |  | 0.09  (-0.22, 0.41) | 0.553 |  | 0.12  (-0.24, 0.48) | 0.508 |

**Supplementary Table 11 Baseline characteristics of the morning and afternoon groups among male participants**

|  | Mean (SD)/ N (%) | | | | |
| --- | --- | --- | --- | --- | --- |
|  | Total | Morning | Afternoon | *P* | |
| N | 146 | 73 | 73 |  | |
| Age | 64.6 (7.0) | 64.5 (7.3) | 64.6 (6.8) | 0.921 | |
| 50-60 years old | 55 (37.7) | 28 (38.6) | 27 (37.0) | 1.000 | |
| 65-75 years old | 91 (62.3) | 45 (61.6) | 46 (63.0) |  |  |
| Smoking | | | | | |
| No | 94 (64.4) | 48 (65.8) | 46 (63.0) | 0.966 | |
| Past smoker | 14 (9.6) | 7 (9.6) | 7 (9.6) |  |  |
| Current smoker | 38 (26.0) | 18 (24.7) | 20 (27.4) |  |  |
| Alcohol consumption | | | | | |
| No | 98 (67.1) | 51 (69.9) | 47 (64.4) | 0.597 | |
| Yes | 48 (32.9) | 22 (30.1) | 26 (35.6) |  |  |
| Hypertension | | | | |  |
| No | 91 (62.3) | 47 (64.4) | 44 (60.3) | 0.733 | |
| Yes | 55 (37.7) | 26 (35.6) | 29 (39.7) |  |  |
| Diabetes | | | | | |
| No | 123 (84.3) | 60 (82.2) | 63 (86.3) | 0.650 | |
| Yes | 23 (15.8) | 13 (17.8) | 10 (13.7) |  |  |
| Coronary heart disease | | | | | |
| No | 132 (90.4) | 68 (93.2) | 64 (87.7) | 0.400 | |
| Yes | 14 (9.6) | 5 (6.9) | 9 (12.3) |  |  |
| History of Influenza vaccination | | | | | |
| No | 125 (85.6) | 66 (90.4) | 59 (80.8) | 0.156 | |
| Yes | 21 (14.4) | 7 (9.6) | 14 (19.2) |  |  |
| Community distribution |  |  |  |  | |
| Shipai Street | 64 (43.8) | 33 (45.2) | 31 (42.5) | 0.868 | |
| Baiyun Street | 82 (56.2) | 40 (54.8) | 42 (57.5) |  |  |
| BMI | 23.9 (3.0) | 24.6 (2.8) | 23.2 (3.0) | 0.005 | |
| Sleep duration(h) | 7.0 (1.4) | 7.0 (1.5) | 7.1 (1.4) | 0.533 | |
| EQ-5D score | 1.0 (0.0) | 1.0 (0.0) | 1.0 (0.0) | 0.907 | |

Abbreviation: SD: standard deviation; BMI: Body Mass Index.

**Supplementary Table 12 Baseline characteristics of the morning and afternoon groups among female participants**

|  | Mean (SD)/ N (%) | | | | |
| --- | --- | --- | --- | --- | --- |
|  | Total | Morning | Afternoon | *P* | |
| N | 243 | 122 | 121 |  | |
| Age | 61.8 (7.1) | 62.0 (7.4) | 61.6 (6.7) | 0.647 | |
| 50-60 years old | 136 (56.0) | 69 (56.6) | 67 (55.4) | 0.897 | |
| 65-75 years old | 107 (44.0) | 53 (43.4) | 54 (44.6) |  |  |
| Smoking | | | | | |
| No | 240 (98.8) | 120 (98.4) | 120 (99.8) | 1.000 | |
| Current smoker | 3 (1.2) | 2 (1.6) | 1 (0.8) |  |  |
| Alcohol consumption | | | | | |
| No | 239 (98.4) | 120 (98.4) | 119 (98.4) | 1.000 | |
| Yes | 4 (1.7) | 2 (1.6) | 2 (1.7) |  |  |
| Hypertension | | | | |  |
| No | 153 (63.0) | 70 (57.4) | 83 (68.6) | 0.084 | |
| Yes | 90 (37.0) | 52 (42.6) | 38 (31.4) |  |  |
| Diabetes | | | | | |
| No | 212 (87.2) | 101 (82.8) | 111 (91.7) | 0.053 | |
| Yes | 31 (12.8) | 21 (17.2) | 10 (8.3) |  |  |
| Coronary heart disease | | | | | |
| No | 224 (92.2) | 111 (91.0) | 113 (93.4) | 0.634 | |
| Yes | 19 (7.8) | 11 (9.0) | 8 (6.6) |  |  |
| History of Influenza vaccination | | | | | |
| No | 194 (79.8) | 102 (83.6) | 92 (76.0) | 0.153 | |
| Yes | 49 (20.2) | 20 (16.4) | 29 (24.0) |  |  |
| Community distribution |  |  |  |  | |
| Shipai Street | 129 (53.1) | 67 (54.9) | 62 (51.2) | 0.608 | |
| Baiyun Street | 114 (46.9) | 55 (45.1) | 59 (48.8) |  |  |
| BMI | 23.8 (3.1) | 23.9 (3.1) | 23.6 (3.0) | 0.781 | |
| Sleep duration(h) | 7.0 (1.3) | 7.0 (1.4) | 7.0 (1.2) | 0.897 | |
| EQ-5D score | 1.0 (0.0) | 1.0 (0.0) | 1.0 (0.0) | 0.308 | |

Abbreviation: SD: standard deviation; BMI: Body Mass Index.

**Supplementary Table 13 The antibody titers prevaccination and post-vaccination of male subgroup**

|  |  |  | Morning（N=73） | Afternoon（N=73） | *P* |
| --- | --- | --- | --- | --- | --- |
| Prevaccination | A/H1N1 | Mean (SD) | 11.1 (8.3) | 17.8 (40.8) | 0.238 |
|  |  | gMean (95%CI) | 10.4 (9.8, 11.0) | 11.5 (10.1, 13.2) |  |
|  |  | Median (IQR) | 10.0 (10.0, 10.0) | 10.0 (10.0, 10.0) |  |
|  | A/H3N2 | Mean (SD) | 58.0 (46.5) | 47.4 (30.9) | 0.151 |
|  |  | gMean (95%CI) | 45.7 (38.8, 53.8) | 38.5 (32.9, 45.1) |  |
|  |  | Median (IQR) | 40.0 (40.0, 80.0) | 40.0 (20.0, 80.0) |  |
|  | B/Victoria | Mean (SD) | 10.0 (0.0) | 10.3 (1.6) | 0.156 |
|  |  | gMean (95%CI) | 10.0 (10.0, 10.0) | 10.2 (9.9, 10.5) |  |
|  |  | Median (IQR) | 10.0 (10.0, 10.0) | 10.0 (10.0, 10.0) |  |
| Post-vaccination | A/H1N1 | Mean (SD) | 72.5 (97.0) | 100.4 (125.6) | 0.485 |
|  |  | gMean (95%CI) | 30.4 (22.3, 41.3) | 36.4 (25.7, 51.6) |  |
|  |  | Median (IQR) | 10.0 (10.0, 80.0) | 10.0 (10.0, 160.0) |  |
|  | A/H3N2 | Mean (SD) | 111.2 (84.6) | 104.3 (84.8) | 0.325 |
|  |  | gMean (95%CI) | 86.3 (72.7, 102.5) | 77.8 (64.8, 93.3) |  |
|  |  | Median (IQR) | 80.0 (80.0, 160.0) | 80.0 (40.0, 160.0) |  |
|  | B/Victoria | Mean (SD) | 33.6 (54.7) | 31.9 (51.2) | 0.636 |
|  |  | gMean (95%CI) | 17.5 (14.0, 21.9) | 17.2 (13.8, 21.4) |  |
|  |  | Median (IQR) | 10.0 (10.0, 20.0) | 10.0 (10.0, 40.0) |  |
| Fold change | A/H1N1 | Mean (SD) | 6.84 (9.26) | 8.79 (11.79) | 0.919 |
|  |  | gMean (95%CI) | 2.92(2.17, 3.95) | 3.15(2.25, 4.43) |  |
|  |  | Median (IQR) | 1.00(1.00, 8.00) | 1.00(1.00, 16.00) |  |
|  | A/H3N2 | Mean (SD) | 3.33 (5.34) | 3.12 (3.44) | 0.703 |
|  |  | gMean (95%CI) | 1.89(1.50, 2.38) | 2.02(1.63, 2.50) |  |
|  |  | Median (IQR) | 2.00(1.00, 4.00) | 2.00(1.00, 4.00) |  |
|  | B/Victoria | Mean (SD) | 3.36 (5.47) | 3.13 (5.09) | 0.543 |
|  |  | gMean (95%CI) | 1.75(1.40, 2.19) | 1.69(1.35, 2.10) |  |
|  |  | Median (IQR) | 1.00(1.00, 2.00) | 1.00(1.00, 4.00) |  |

Abbreviation: SD: standard deviation; IQR: interquartile range; gMean: geometric mean; CI: confidence interval.

**Supplementary Table 14 The antibody titers prevaccination and post-vaccination of female subgroup**

|  |  |  | Morning（N=122） | Afternoon（N=121） | *P* |
| --- | --- | --- | --- | --- | --- |
| Prevaccination | A/H1N1 | Mean (SD) | 11.9 (15.0) | 10.9 (7.0) | 1.000 |
|  |  | gMean (95%CI) | 10.5 (9.9, 11.1) | 10.4 (9.9, 10.8) |  |
|  |  | Median (IQR) | 10.0 (10.0, 10.0) | 10.0 (10.0, 10.0) |  |
|  | A/H3N2 | Mean (SD) | 46.1 (27.0) | 46.6 (28.5) | 0.868 |
|  |  | gMean (95%CI) | 39.1 (35.2, 43.5) | 38.9 (34.7, 43.6) |  |
|  |  | Median (IQR) | 40.0 (20.0, 80.0) | 40.0 (40.0, 40.0) |  |
|  | B/Victoria | Mean (SD) | 10.1 (0.9) | 10.3 (2.7) | 1.000 |
|  |  | gMean (95%CI) | 10.1 (9.9, 10.2) | 10.1 (9.9, 10.4) |  |
|  |  | Median (IQR) | 10.0 (10.0, 10.0) | 10.0 (10.0, 10.0) |  |
| Post-vaccination | A/H1N1 | Mean (SD) | 122.4 (133.9) | 93.1 (126.0) | 0.030 |
|  |  | gMean (95%CI) | 46.9 (35.6, 61.8) | 31.1 (23.8, 40.7) |  |
|  |  | Median (IQR) | 40.0 (10.0, 320.0) | 10.0 (10.0, 160.0) |  |
|  | A/H3N2 | Mean (SD) | 128.1 (99.5) | 111.2 (90.6) | 0.176 |
|  |  | gMean (95%CI) | 96.0 (83.5, 110.3) | 84.7 (74.4, 96.5) |  |
|  |  | Median (IQR) | 80.0 (40.0, 160.0) | 80.0 (40.0, 160.0) |  |
|  | B/Victoria | Mean (SD) | 27.5 (51.5) | 22.3 (42.8) | 0.061 |
|  |  | gMean (95%CI) | 14.8 (12.7, 17.3) | 13.0 (11.3, 14.9) |  |
|  |  | Median (IQR) | 10.0 (10.0, 10.0) | 10.0 (10.0, 10.0) |  |
| Fold change | A/H1N1 | Mean (SD) | 11.93 (13.34) | 9.23 (12.63) | 0.036 |
|  |  | gMean (95%CI) | 4.48(3.40, 5.90) | 3.00(2.29, 3.94) |  |
|  |  | Median (IQR) | 4.00(1.00, 32.00) | 1.00(1.00, 16.00) |  |
|  | A/H3N2 | Mean (SD) | 3.92 (5.35) | 3.30 (4.08) | 0.250 |
|  |  | gMean (95%CI) | 2.45(2.08, 2.89) | 2.18(1.87, 2.54) |  |
|  |  | Median (IQR) | 2.00(1.00, 4.00) | 2.00(1.00, 4.00) |  |
|  | B/Victoria | Mean (SD) | 2.74 (5.15) | 2.13 (4.09) | 0.082 |
|  |  | gMean (95%CI) | 1.47(1.26, 1.72) | 1.28(1.13, 1.46) |  |
|  |  | Median (IQR) | 1.00(1.00, 1.00) | 1.00(1.00, 1.00) |  |

Abbreviation: SD: standard deviation; IQR: interquartile range; gMean: geometric mean; CI: confidence interval.

**Supplementary Table 15 The number of seroconverted individuals and the increase between post- and pre-vaccination GMT titer for male subgroup**

|  |  | Morning(N=73) | Afternoon(N=73) | P-values |
| --- | --- | --- | --- | --- |
| A/H1N1 seroprotected  N (%) | pre-vaccination | 1 (1.37) | 4 (5.48) | 0.366 |
|  | post-vaccination | 33 (45.21) | 33 (45.21) | 1.000 |
| A/H1N1 seroconverted  N (%) | pre-vaccination | 2 (2.74) | 6 (8.22) | 0.275 |
|  | post-vaccination | 38 (52.05) | 35 (47.95) | 0.741 |
| A/H3N2 seroprotected  N (%) | pre-vaccination | 59 (80.82) | 51 (69.86) | 0.178 |
|  | post-vaccination | 70 (95.89) | 68 (93.15) | 0.719 |
| A/H3N2 seroconverted  N (%) | pre-vaccination | 70 (95.89) | 68 (93.15) | 0.719 |
|  | post-vaccination | 72 (98.63) | 72 (98.63) | 1.000 |
| B/Victoria seroprotected  N (%) | pre-vaccination | 0(0.00) | 0(0.00) | / |
|  | post-vaccination | 16 (21.92) | 20 (27.40) | 0.565 |
| B/Victoria seroconverted  N (%) | pre-vaccination | 0 (0.00) | 3 (4.11) | 0.245 |
|  | post-vaccination | 28 (38.36) | 25 (34.25) | 0.731 |

**Supplementary Table 16 The number of seroprotected and seroconverted individuals pre- and post-vaccination for female subgroup**

|  |  | Morning(N=122) | Afternoon(N=121) | P-values |
| --- | --- | --- | --- | --- |
| A/H1N1 seroprotected N (%) | pre-vaccination | 2 (1.64) | 2 (1.65) | 1.000 |
|  | post-vaccination | 64 (52.46) | 47 (38.84) | 0.039 |
| A/H1N1 seroconverted N (%) | pre-vaccination | 7 (5.74) | 6 (4.96) | 1.000 |
|  | post-vaccination | 67 (54.92) | 49 (40.50) | 0.029 |
| A/H3N2 seroprotected N (%) | pre-vaccination | 90 (73.77) | 95 (78.51) | 0.452 |
|  | post-vaccination | 118 (96.72) | 118 (97.52) | 1.000 |
| A/H3N2 seroconverted N (%) | pre-vaccination | 116 (95.08) | 110 (90.91) | 0.220 |
|  | post-vaccination | 121 (99.18) | 120 (99.17) | 1.000 |
| B/Victoria seroprotected N (%) | pre-vaccination | 0 (0.00) | 1 (0.83) | 0.498 |
|  | post-vaccination | 19 (15.57) | 13 (10.74) | 0.343 |
| B/Victoria seroconverted N (%) | pre-vaccination | 1 (0.82) | 1 (0.83) | 1.000 |
|  | post-vaccination | 32 (26.23) | 21 (17.36) | 0.120 |

**Supplementary Table 17 The occurrence of adverse events in the morning and afternoon group**

|  | Morning  (N=204) | Afternoon  (N=201) |
| --- | --- | --- |
| Overall | 14 (6.9%) | 13 (6.5%) * |
| Fatigue | 3 (1.5%) | 4 (2.0%) |
| Sweating | 1 (0.5%) | 1 (0.5%) |
| Fever | 1 (0.5%) | 0 (0.0%) |
| Runny nose | 3 (1.5%) | 1 (0.5%) |
| Cough | 0 (0.0%) | 1 (0.5%) |
| Sore throat | 0 (0.0%) | 1 (0.5%) |
| Nasal obstruction | 0 (0.0%) | 1 (0.5%) |
| Rash | 1 (0.5%) | 1 (0.5%) |
| Vertigo | 1 (0.5%) | 2 (1.0%) |
| Myalgia | 2 (1.0%) | 3 (1.5%) |
| Headache | 1 (0.5%) | 1 (0.5%) |
| Chill | 0 (0.0%) | 1 (0.5%) |
| Palpitation | 1 (0.5%) | 0 (0.0%) |
| Hand numbness | 0 (0.0%) | 1 (0.5%) |
| Lymphadenectasis | 0 (0.0%) | 1 (0.5%) |
| Vomiting | 0 (0.0%) | 1 (0.5%) |
| Diarrhea | 1 (0.5%) | 1 (0.5%) |

*14 patients occurred 15 adverse events in Morning and 13 patients occurred 21 adverse events in afternoon group, p=1.000.


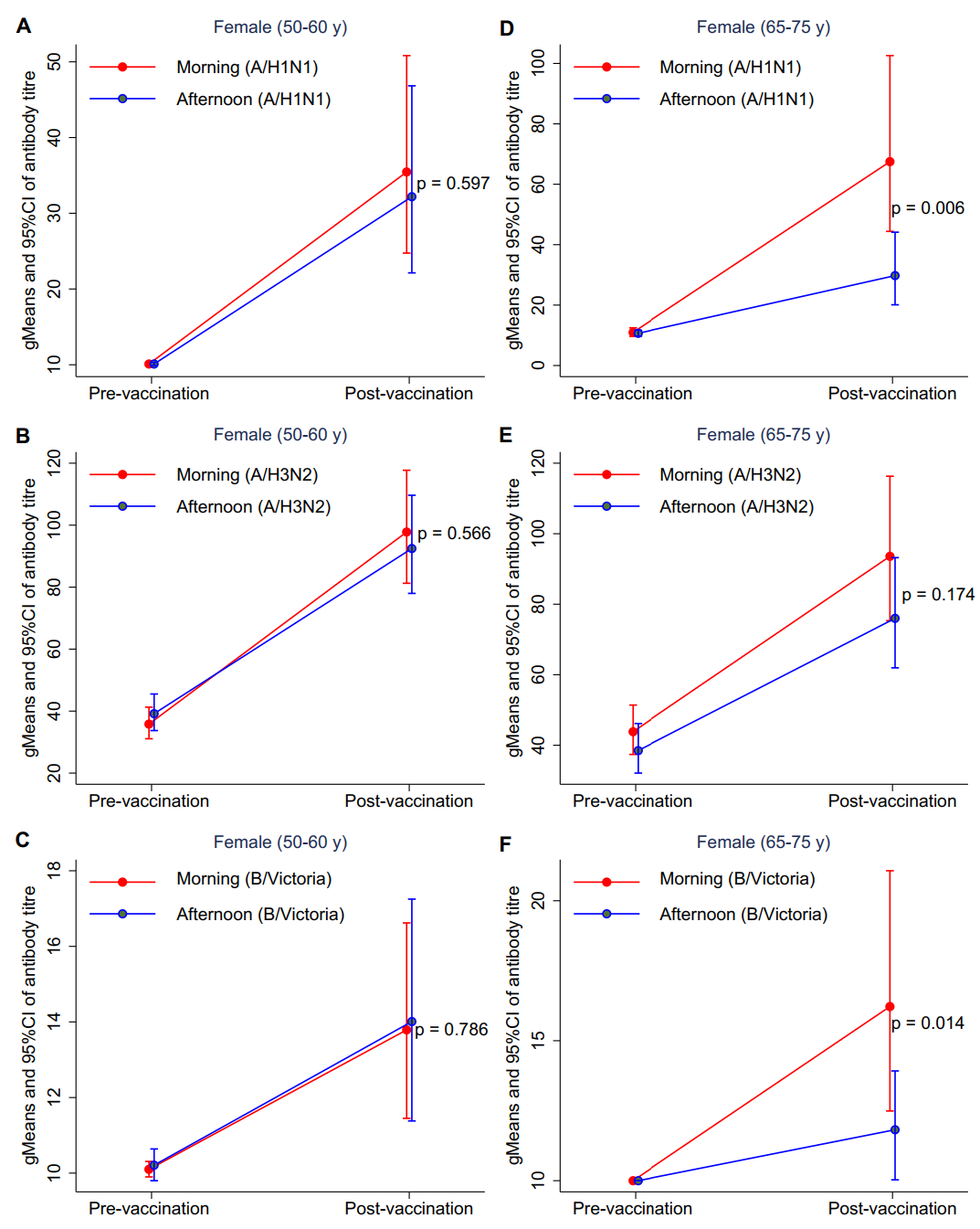


**Supplementary Figure 1 Antibody titers prevaccination and post-vaccination for females by age**

A-C: The geometric mean (95% CI) for antibody titers of A/H1N1, A/H3N2 and B/Victoria strain for females aged 50-60

D-F：The geometric mean (95% CI) for antibody titers of A/H1N1, A/H3N2 and B/Victoria strain for females aged over 65


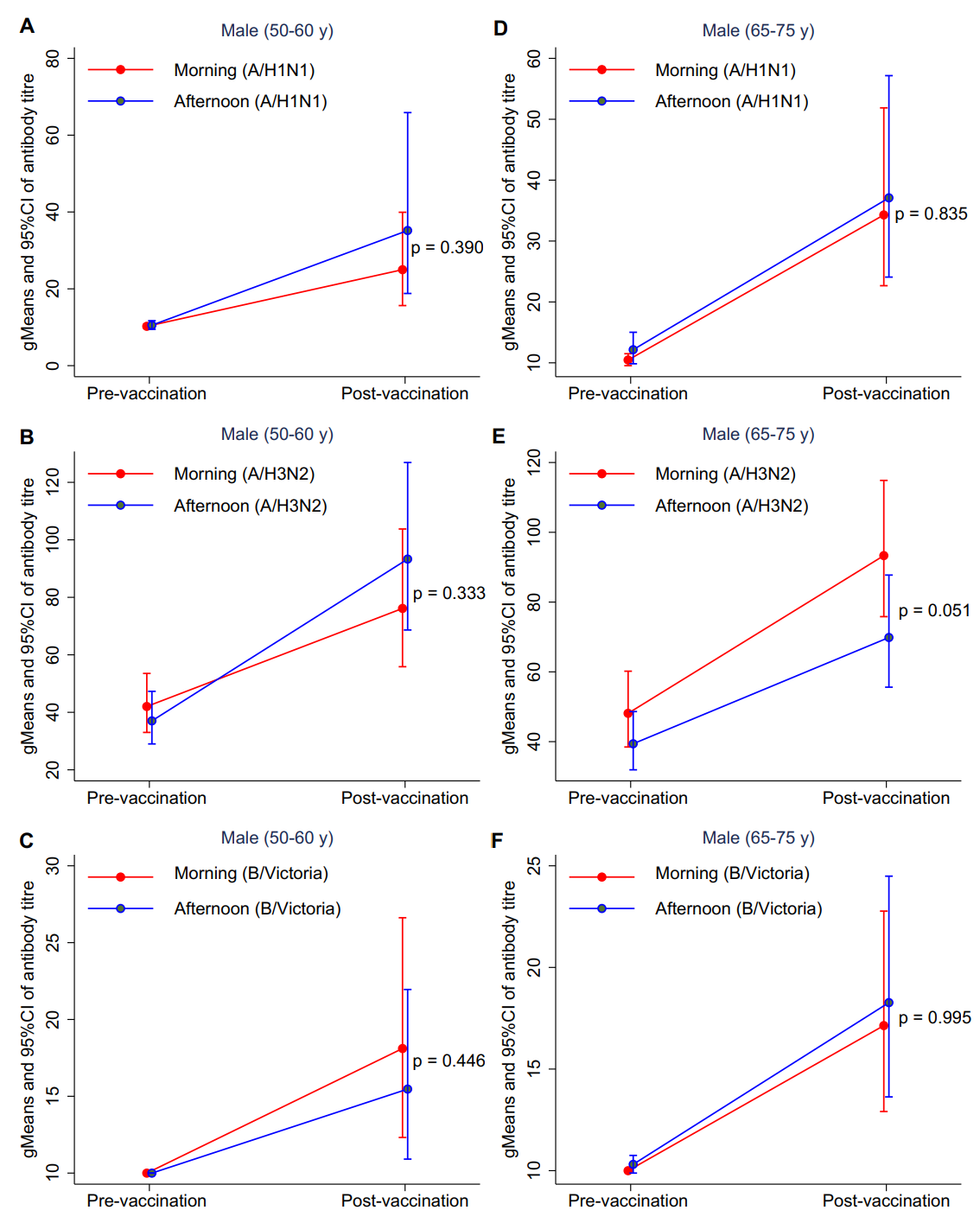


**Supplementary Figure 2 Antibody titers prevaccination and post-vaccination for males by age**

A-C: The geometric mean (95% CI) for antibody titers of A/H1N1, A/H3N2 and B/Victoria strain for males aged 50-60

D-F：The geometric mean (95% CI) for antibody titers of A/H1N1, A/H3N2 and B/Victoria strain for males aged over 65
